# Association between endocrine therapy and cognitive decline in breast cancer based on propensity score matching

**DOI:** 10.3389/fmed.2023.1132287

**Published:** 2023-01-26

**Authors:** Yulian Yin, Lan Jin, Meiling Chu, Yue Zhou, Siyuan Tu, Yifan Cheng, Meina Ye, Jingjing Wu, Hongfeng Chen

**Affiliations:** Longhua Hospital Shanghai University of Traditional Chinese Medicine, Shanghai, China

**Keywords:** breast cancer, endocrine therapy, cognitive function, association, propensity score matching

## Abstract

**Purpose:**

To study the *status quo* of the cognitive function of the breast cancer patients with (who went through) the endocrine therapy by the epidemiological investigation, analyze the key factor of the cognition impairment and explore the impact of the endocrine therapy time on the cognition decline after using Propensity Score Matching to balance the covariates.

**Methods:**

In this study, the epidemiological questionnaire information was collected from 226 female breast cancer endocrine treatment patients who visited the Breast Clinic of Longhua Hospital Affiliated to Shanghai University of Chinese Medicine from November 2020 to February 2022, and the results of the overall cognitive function, the function test of each cognitive domain, the patient’s self-cognition, quality of life, and emotional status evaluation of the patients. In this study, according to the principle of random matching, the nearest matching method with a matching tolerance of 0.2 and a matching ratio of 1:2 was used for orientation score matching. After the covariant such as age, BMI, and duration of education were balanced, the effects of the duration of endocrine therapy on the overall cognitive function and the functions of each cognitive domain were analyzed.

**Results:**

In 226 cases of female breast cancer patients (who went through) the endocrine therapy, the propensity score matching was performed, ultimately, 99 were ruled out, successful matched ones were 49 of the cognition-decline group and 78 of the standard group. With age, education time, BMI and other covariates balanced, the endocrine therapy duration was the risk factor of the cognition impairment (*P* < 0.05, OR = 1.296, 95% CI = 1.008−1.665), with the extension of endocrine treatment time, there was a rising risk of the cognition impairment (LLA statistic = 5.872, *P* < 0.05). The cognitive domain scores in the cognition-decline group were lower than the standard group (*P* < 0.05), but there was a difference in self-report cognition.

**Conclusion:**

The endocrine therapy duration was the risk factor for the cognition impairment of the breast cancer patients, and with prolonged endocrine treatment, there was a rising (an increasing) risk for the cognition impairment.

## Introduction

According to the statistics of the International Agency for Research on Cancer, in 2020, female breast cancer ranked first in the incidence of malignant tumors in the world, accounting for 11.7% of all new cases of malignant tumors (1). There were 416,371 new cases of breast cancer in China, accounting for 19.9% of the total number of new female cancers, and ranking first in the incidence of female cancers in China. With the increasing level of diagnosis and treatment of breast cancer, the five-year survival rate has reached 90% (2). About 70% of patients with breast cancer are hormone receptor positive (3). Endocrine therapy is a special and long-term treatment that needs to be received by hormone receptor positive breast cancer patients. To receive endocrine therapy for 5–10 years is currently recognized as the first-line plan to prevent tumor recurrence and prolong the survival time of patients. In view of the high incidence and relatively high survival rate of breast cancer, the quality of life of patients has become a close concern. According to the statistics of some scholars, the common symptoms of breast cancer patients receiving endocrine therapy include climacteric symptoms, bone-related adverse events, lymphedema, fatigue, depression, sleep disorders and cognitive impairment, among which the related research on cognitive impairment is rare (4). Endocrine therapy for breast cancer is an anti-tumor regimen targeting the resistance to estrogen, which is able to protect the nervous system and plays an important role in improving the cognitive function and memory ability of women (5). All of the above have suggested the possibility of cognitive impairment in breast cancer patients receiving endocrine therapy, but the relationship between the two is still unclear (6). In this study, we used the propensity score matching method to balance various variables that differ between the cognitive decline and the normal in endocrine patients with breast cancer, making the duration of endocrine treatment the only test variable to assess the impact of the duration of endocrine treatment on cognitive function.

### Study design

**FIGURE 1:**
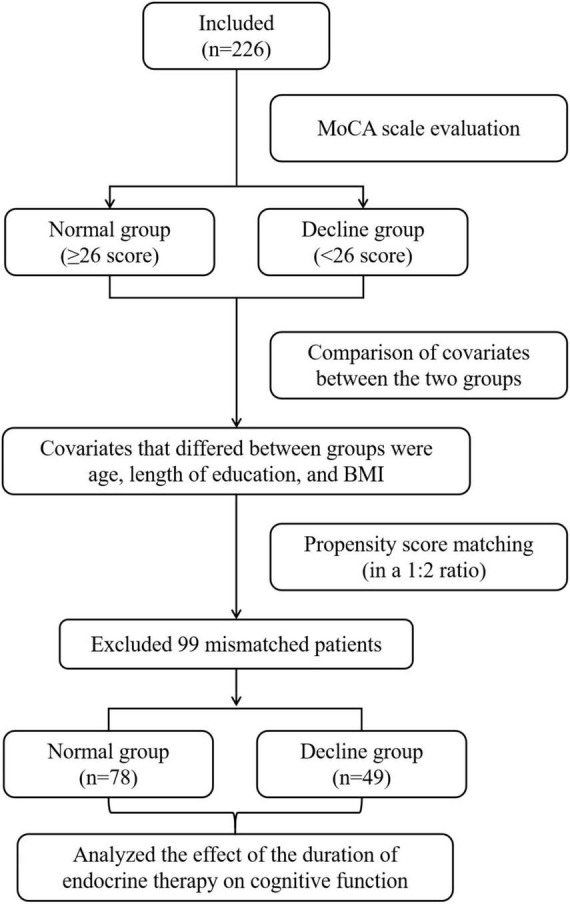
Schematic diagram of the research flowchart.

This study is a prospective interventional single-center study. The subjects of this study were breast cancer patients who were treated in the first department of breast cancer clinic of Longhua Hospital affiliated to Shanghai University of Traditional Chinese Medicine from November 2020 to February 2022. A schematic diagram of the research flowchart is shown in Figure 1.

The Medical Ethics Committee of Longhua Hospital affiliated to Shanghai University of Traditional Chinese Medicine approved this research. Written informed consent was obtained from all individual participants included in the study. The clinical trial registration number is ChiCTR2200057785.

### Participant

All participants met the 2020 Guidelines for Diagnosis and Treatment of Breast Cancer by the Chinese Society of Clinical Oncology (CSCO) (7), and were clearly diagnosed with breast cancer based on basic and molecular pathology. Inclusion criteria: (1) Patients were diagnosed with breast cancer, and the immunohistochemical examination of postoperative pathology showed positive ER and/or PR. (2) Endocrine therapy lasting for three months or more, including selective hormone receptor modulators, and/or aromatase inhibitors, and/or ovarian castration. (3) Female patients aged 30–55 years old (including 30 and 55 years old). Whisenant et al. (4) have enough visual, auditory, and cultural degree, can read and write simple sentences. (4) Individual oral communication can be conducted and blood tests can be completed with coordination. (5) Understand and voluntarily participate in project research and sign informed consent form. Exclusion criteria: (1) Patients were under treatment for breast cancer recurrence and/or metastasis. (2) Selective hormone receptor modulators, and/or aromatase inhibitors, and/or ovarian castration have been used before breast cancer. (3) The cerebrovascular diseases or neurodegenerative diseases that affect cognitive function, such as vascular dementia, Alzheimer’s disease, and frontotemporal dementia, are confirmed. (4) Anxiety, depression, schizophrenia and other mental disorders were confirmed by the specialized hospital.

## Materials and methods

### Observation indexes

1) Overall cognitive function scale: Montreal Cognitive Assessment (MoCA).

2) Sub-cognitive domain function scale(memory, language ability, attention, visual space and execution function):

➀ Memory test scale: Auditory verb learn test-Huashan version (AVLT-H).

➁ Language ability test scale: Verbal fluency test (VFT).

➂ Attention test scale: Digital span test (DST).

➃ Visual space and executive ability test scale: Trail Making Test (TMT-A, TMT-B) and clock-drawing test (CDT).

3) Self-rating cognitive scale: Functional Assessment of Cancer Therapy-Cognitive Function (FACT-cog) (8). The FACT-cog scale consists of four factor scales, namely, perceived cognitive impairments (CogPCI), perceived cognitive abilities (CogPCA), and others’ comments. Co goal) and the impact of cognitive changes on quality of life (CogQOL).

4) Examination indicators: Serum Estradiol (E2) and Follicle-stimulating Hormone (FSH); Test time point: the test was performed on the third day of the first menstrual cycle of patients without menopause before or after the investigation; Patients who have already stopped menstruating are tested before investigation or within 1 week after investigation.

### Correction and matching of samples

Previously, we completed the overall cognitive function evaluation of 226 patients using convenient sampling. The score of MoCA scale <26 was judged as overall cognitive function decline, and a total of 75 patients (33.19%) were included. We compared the basic information of the subjects with normal cognitive function (normal group) and those with decreased overall cognitive function (decline group) in the overall sample, and found that there were statistical differences in age, educational duration and BMI of the two groups of people, as shown in Appendix Table 1, which might mask the effect of the duration of endocrine therapy on cognitive function. Therefore, we included the patient’s age, BMI, and educational duration in the covariates for tendency score matching, and used PSM extension of SPSS 22.0 software for tendency score matching, so that the factors of uneven distribution between groups were balanced.

**FIGURE 2:**
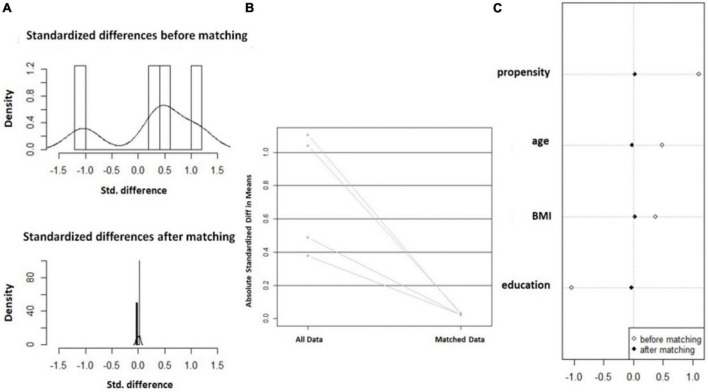
Balance change diagram before and after orientation score matching. **(A)** Histogram of SD; **(B)** Standardized mean difference line diagram; and **(C)** Scatter plot of single variable SD.

In this study, based on the principle of random matching, the closest matching method with the matching tolerance (Caliper) of 0.2 and the matching ratio of 1:2 between two groups of samples was used for orientation score matching. After 99 mismatched cases were excluded, 78 cases in the normal group and 49 cases in the decline group were successfully matched. The statistical value of L1 measure after matching is 0.724, which is less than 0.781 before matching. There is no variable | d | d| >0.25, which indicates that the matching is good and all the matched variables are balanced. The absolute values of the standard differences in age, BMI and educational duration after matching decreased from 48.6, 37.7, and 103.8% before matching to 2.2, 2.4, and 3.1% after matching, respectively, all less than 10%, suggesting that the inter-group balance was good after matching, as shown in Figure 2.

### Quality control

The investigator strictly followed the inclusion and exclusion criteria of clinical trial protocol to select the appropriate subjects and conducted the investigation and data entry as required. To reduce the random error caused by different researchers in the scale investigation, all scale investigations for subjects were conducted by the investigating doctor himself/herself, and the data of subjects were sequenced and numbered according to the time sequence of completing the investigation. Other researchers regularly examined the subject’s data, checked whether the case report form was correctly filled, whether there were omissions, signatures, and dates, and so on, and fed back the problems to the investigating doctor for correction after they found them.

### Data management

Within 1 week after the completion of the investigation, all the data in the case report form were entered into the Excel table relevant to this study, and 20% of the data were extracted for manual verification again. Any error entered was corrected based on the original data.

### Statistical methods

Statistical analysis was performed using SPSS 22.0 software. All tests were bilateral, and the test level was α = 0.05. The level of statistical significance was set at *P* < 0.05. Descriptive statistics were performed for each variable. First, normality test was performed for measurement data. The results were expressed as mean standard deviation (x̄ s) for normal distribution, such as age and BMI. If the data did not follow the normal distribution, the results were expressed as median and quartile *M*(*P*_25_, *P*_75_), such as menarche age. Independent sample t test was used to test the difference between the two groups of variables that were in accordance with the normal distribution and with uniform variance, such as the age of the two groups after matching, and Wilcoxon rank sum test was used for the rest, such as the duration of endocrine treatment in the two groups. The enumeration data or classified variables were expressed as frequency and percentage. For example, the number of patients with or without family history of cancer and the classification of endocrine protocol were included. The difference between the two groups of variables was examined by χ^2^ test or Fisher exact probability method.

The OR value and 95% confidence interval of the risk relationship between the duration of endocrine treatment and the cognitive decline of endocrine treatment patients were calculated by conditional logistic regression analysis. The Linear-by-Linear Association test (LLA) (9) was used to analyze the trend relationship between the duration of endocrine therapy and the risk of cognitive decline.

## Results

### Patient characteristics

After matching, the covariates such as age, education duration, BMI, tumor family history, basic disease, and medication history, pregnancy duration, lactation duration, menopausal status, menarche age, tumor treatment (chemotherapy, radiotherapy, targeted therapy, endocrine medication type), emotional state (SAS and SDS), quality of life, and TCM symptom score of the normal group and the declined group were balanced and comparable, as shown in Appendix Table 1.

### Correlation between duration of endocrine therapy and overall cognitive decline

**FIGURE 3:**
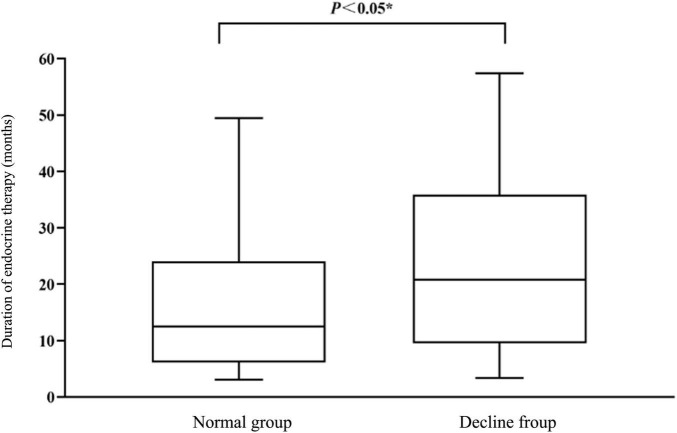
Comparison of endocrine therapy duration (months) between normal group and declining group.

After normality test, the duration of endocrine treatment did not follow the normal distribution. The duration of endocrine treatment in the descending group was 20.77(9.52, 35.88) months, which was significantly higher than that of 12.5(6.09, 24.07) months in the normal group. The statistical value z = −2.434 and *P* = 0.015 < 0.05 by non-parametric test were obtained, and the difference was statistically significant, as shown in Figure 3.

The duration of endocrine treatment was divided into six groups and conditional logistic regression analysis was performed. The negative double likelihood pair value after the formal inclusion of the variable was 87.084, which was less than the negative double likelihood pair value of the invalid model was 91.445, suggesting that the effect of the model with the included variable was better than that of the invalid model. Moreover, χ^2^ = 4.290 and *P* = 0.038 indicated that the conditional Logistic regression was statistically significant. The results of conditional logistic regression analysis showed that the duration of endocrine treatment was positively correlated with the overall cognitive decline. The duration of endocrine treatment was a risk factor for the overall cognitive decline (*P* < 0.05, OR = 1.296, 95% CI = 1.008−1.665), as shown in Table 1. At the same time, the results of the LLA trend test suggested that the risk of overall cognitive decline increased with the prolongation of endocrine therapy (LLA statistic = 5.872, *P* = 0.015 < 0.05).

**TABLE 1:** Comparison of duration of endocrine treatment in normal and decline groups.

Endocrine treatment duration/Month	Normal group, *n* = 78(%)	Decline group, *n* = 49(%)
3−6	19(24.36)	6(12.25)
6−12	18(23.08)	10(20.41)
12−24	22(28.21)	12(24.49)
24−36	10(12.82)	9(18.37)
36−48	6(7.69)	7(14.28)
48−60	3(3.84)	5(10.20)
Statistic	Wald χ^2^ = 4.104, *P* = 0.043*	
OR(95%CI)	1.296(1.008−1.665)	

*Stands for statistical difference.

### Comparison of sub-cognitive domain functions (memory, language ability, attention, visual space, and executive function) between the two groups

As shown in Table 2, the scores of short time, short delay memory, long delay memory, and memory and recognition after cue presentation in the decline group were lower than those of the normal group. In the language function module, we used the 60−s word fluency test, which was based on the 15−s cutoff score. The word output and total score of patients in the decline group were lower than those in the normal group for 15 s. In the DST of reactive attention level, the anteroposterior, inversion and total scores of the decline group were lower than those of the normal group. In the Visual Space and Executive Function evaluation module, the TMT and CDT test scores were lower in the decline group than in the normal group. There was no statistical difference in the subjective evaluation of cognitive function between the two groups.

**TABLE 2:** Comparison of sub-cognitive domain function between normal group and decline group.

	Item	Normal group	Decline group	Statistic	*P*-value
Memory module; AVLT-H	AVLT short-term memory	21.85 ± 3.26	18.27 ± 3.32	T = −5.987	0.000
	AVLT short delay recall	8(7, 10)	6(5, 8)	Z = −5.245	0.000
	AVLT long delay recall	8(6, 9)	6(5, 8)	Z = −4.843	0.000
	AVLT cued recall	8(7, 10)	6(5, 8)	Z = −4.243	0.000
	AVLT recognition	11(10, 12)	10(9, 11)	Z = −2.801	0.005
language ability; VFT	VFT	20.6 ± 4.54	16.78 ± 3.53	T = −5.021	0.000
	VFT-1st	10(9, 12)	9(8, 10)	Z = −2.357	0.018
	VFT-2nd	5(3, 6)	3(2, 5)	Z = −3.478	0.001
	VFT-3rd	3(1, 4)	2(1, 3)	Z = −2.828	0.005
	VFT-4th	3(2, 3.25)	2(1, 3)	Z = −2.411	0.016
Attention module; DST	Forward Digit Span	12(10, 13)	10(7.5, 12)	Z = −3.691	0.000
	Backward Digit Span	8(6, 10.25)	7.22 ± 2.49	Z = −2.669	0.008
	Total Score	19.5(17, 2.25)	17.04 ± 4.45	Z = −3.568	0.000
Visual space and Executive ability module; TMT-A TMT-B CDT	TMT-A	32.32 ± 8.18	37(31, 42)	Z = −5.245	0.000
	TMT-B	72.5(60, 95.25)	97(72.5, 131.5)	Z = −3.472	0.001
	TMT interference*	40(25.75, 65.25)	62(41, 9)	Z = −2.908	0.004
	CDT	10(8, 10)	7(6.5, 9.5)	Z = −4.591	0.000
Self-rating cognitive module; FACT-cog	CogPCI	59.6 ± 14.28	63(53.5, 69)	Z = −0.513	0.608
	CogPCA	21.53 ± 7.45	22(16, 27)	Z = −0.404	0.686
	CogOth	16(14, 16)	16(14, 16)	Z = −0.501	0.616
	CogQOL	14(11.75, 16)	14(11, 16)	Z = −0.961	0.337
	FACT-cog	109.5 ± 22.16	113(92.5, 123)	Z = −0.528	0.598

*The TMT-B completion time minus the TMT-A completion time is the TMT interference amount.

### Comparison of serum estradiol and follicle stimulating hormone levels between the two groups

There was no statistical difference in serum estradiol and follicle-stimulating hormone levels between the normal group and the decline group, as shown in Table 3.

**TABLE 3:** Comparison of E_2_ (pg/ml) and FSH (IU/L) values between the normal group and the decline group.

	E_2_	FSH
Normal group	10.08(6.06, 28.81)	10.36(5.82, 28.79)
Decline group	10.08(10.08, 22.17)	13.58(6.33, 25.02)
Statistic	Z = −0.809, *P* = 0.418	Z = −0.461, *P* = 0.645

## Discussion

The concept of tendency scoring was first proposed by statistical experts Rosenbaum and Rubin in 1983 (10), and the theoretical basis and dimension reduction process of tendency scoring were elaborated in detail. It is a powerful tool to deal with the confounding bias in observational studies and has a wide application prospect (11). Among them, Propensity Score Matching (PSM) is the most commonly used one, which matches the individuals with the same or similar propensity scores in the treatment group and the control group in 1:1 or 1:n to make the covariates of the two groups in a balanced state. The processing effect is estimated based on the matched data sets, which has the advantage of ensuring the objectivity of the study (12). Moreover, when the sample size is 200, the relative bias of the propensity score matching is the smallest (13).

Huang et al. (14) used PSM to match the rehabilitation patients after a tibial plateau operation in two groups and concluded that comprehensive rehabilitation treatment with traditional Chinese medicine could improve the rehabilitation efficacy of tibial plateau fracture within six months after the operation. Wang et al. (15) used PSM to balance the inter-group covariates to explore the correlation between serum scandium level and oral cancer risk, and they believed that serum scandium level had a negative correlation with oral cancer risk. PSM is also applied to relevant research on breast cancer. Sun (16) matched the PSM of postmenopausal HR-positive breast cancer patients receiving new adjuvant endocrine therapy with those receiving postoperative adjuvant endocrine therapy, to explore the efficacy and prognosis of postmenopausal HR-positive breast cancer patients receiving new adjuvant endocrine therapy. It was considered that the five-year disease-free survival and total survival of postmenopausal breast cancer patients receiving pre-operative new adjuvant endocrine therapy or post-operative adjuvant endocrine therapy were the same.

In this study, we also used PSM to balance the covariates except for the duration of endocrine therapy to explore the effect of the duration of endocrine therapy on the cognitive function of patients with breast cancer treated by endocrine therapy. We matched the overall cases and found that there was no difference in the self-cognition scores of patients after matching between the two groups with normal or decreased overall cognitive function, and the effects of personal characteristics such as age, educational level, and BMI on the self-cognition of subjects were controlled. The results showed that the duration of endocrine therapy was a risk factor for overall cognitive decline, and with of prolonged endocrine therapy, the risk of overall cognitive decline in breast cancer patients receiving endocrine therapy also increased.

With the changes in clinical practice guidelines for breast cancer, there have been two major changes in endocrine therapy for HR-positive breast cancer patients. First, endocrine drugs are continuously developed and marketed. At first, TAM is the main therapeutic drug, and AIs are later used as the first-line drug for postmenopausal breast cancer patients. The second major change is the extension of treatment time, which ranges from two to three years at first to five to eight years and then to 10 years (17). It is generally believed that extended endocrine therapy can reduce the risk of recurrence and metastasis of breast cancer. At the same time, long-term use of endocrine therapy can cause a series of side effects, such as endometrial lesions, venous thrombosis, and osteoporosis (18, 19), which have been widely reported. At present, some scholars have paid attention to the fact that endocrine therapy may affect the cognitive function of breast cancer patients to a certain extent. Chen et al. (20) focused on detecting the effect of TAM on the executive attention of breast cancer patients and found that patients taking TAM had poorer executive function than patients not taking TAM and healthy people. Liao et al. (21) conducted a case-control trial to explore the association between TAM and Alzheimer’s disease. They compared breast cancer patients over 65 years old with AD and breast cancer patients without any dementia-related diseases and analyzed TAM as the main exposure factor. They found that compared with patients who did not use TAM, patients who had used TAM had an OR value of 3.09 for AD. However, the researchers finally believed that the use of TAM might be a survival effect rather than a toxicity effect to increase the risk of AD. Therefore, the relationship between endocrine therapy and cognitive impairment in breast cancer needs further investigation. In our study, breast cancer patients who have received endocrine therapy for five years are taken as the research object. The research results show that the duration of endocrine therapy is a risk factor for cognitive decline. That is to say, the overall cognitive function of patients tends to decrease with the prolongation of endocrine therapy.

The comparison of the overall decreased cognitive function with that of normal patients in each cognitive domain revealed that patients with overall decreased cognitive function had a certain decrease in the ability scores in each cognitive domain, that is, the prolongation of endocrine therapy not only affected the overall cognition of patients but also caused adverse effects on patients in each cognitive domain dimension. For example, patients with decreased overall cognitive function showed decreased memory in all dimensions (short time, short delay, long delay, clues, and recognition memory). For the influence on language function, the result of the original 60−s language function test can be shortened to 15 s, that is, the 15−s language function test can objectively detect the decline in the language function of patients. In the aspect of attention, DST can be directly used to test attention in the clinical process. The attention of patients in the overall cognitive decline group was lower than that in the normal group. However, the median DST of the two groups of patients was higher than the conventional standard 6 points from the results, suggesting that the cognitive decline of female breast cancer patients aged 30−55 years old receiving endocrine therapy might not be mainly manifested as inattention, or the conventional standard 6 points for people over 60 years old used to evaluate female breast cancer patients aged 30−55 years old might overestimate their attention. From the TMT and CDT test results reflecting visual space and executive ability, the scores of patients in the decline group were lower than those of the normal group in both tests, but the median CDT of the decline group was higher than the conventional 6-point standard, suggesting that for patients with decreased cognitive function, visual space and executive ability should not be taken as the primary impaired cognitive domain.

At the same time, no statistical differences were observed in E_2_ and FSH levels between the baseline-consistent groups. This may be related to the low levels of both E_2_ and FSH in breast cancer patients after endocrine treatment, while the low levels cannot reflect their effects on cognitive function. It is also possible that the objectively detectable levels of E_2_ and FSH in peripheral blood after endocrine treatment cannot reflect the overall situation of sex hormones in patients’ bodies, and cannot reflect their effects on cognitive function.

It should be noted that regardless of whether the overall cognitive function of patients is decreased, there is the uncertainty of over-estimation or under-estimation for their cognitive function, and the results of self-evaluation cognition are not consistent with the measured cognition, which may be a major feature of patients with breast cancer treated by endocrine therapy. Therefore, in the clinical process, patients’ judgment cannot be used as the standard to make clinical diagnoses and treatment decisions.

## Conclusion

To sum up, our results still need a larger cohort dataset and more rigorous research evidence. However, according to the results of this study, prolonged endocrine therapy does have certain adverse effects on cognitive function. Although the current guidelines both in China and abroad advocate extending the duration of endocrine therapy, clinicians need to assess the proportion of risks and benefits in consideration of patients’ conditions to make optimal clinical decisions. Meanwhile, some drugs, rehabilitation, and physical therapy may reduce the risk of cognitive decline caused by endocrine therapy, which of course also needs further research.

## Data availability statement

The original contributions presented in this study are included in the article/supplementary material, further inquiries can be directed to the corresponding authors.

## Ethics statement

The studies involving human participants were reviewed and approved by the Medical Ethics Committee of Longhua Hospital affiliated to Shanghai University of Traditional Chinese Medicine. Written informed consent for participation was not required for this study in accordance with the national legislation and the institutional requirements.

## Author contributions

YY and LJ: conceptualization, formal analysis, data curation, and visualization. LJ: writing—original draft. YY and MC: validation, visualization, writing—review and editing. MY: methodology. YZ, ST, and YC: investigation, validation, and data curation. JW and HC: supervision, project administration, and funding acquisition. All authors contributed to the article and approved the submitted version.

## Appendix

**APPENDIX TABLE A1:** Balance comparisons of covariates between the normal group and the declining group after propensity score matching.

Variable		Before matching				After matching			
		Normal group(*n* = 151)	Decline group(*n* = 75)	Statistic	*P*	Normal group(n = 78)	Decline group(*n* = 49)	Statistic	*P*
Age		46(40, 49)	49(44, 52)	Z = −3.448	0.001*	46.42 ± 5.65	46.96 ± 5.79	T = 0.516	0.607
Length of education		15(13.50, 16)	9(8, 14)	Z = −6.967	0.000*	14(11, 16)	12.51 ± 3.83	Z = −1.329	0.184
BMI		21.67(20.32, 23.61)	22.97 ± 2.42	Z = −2.894	0.004*	22.69 ± 2.92	23.59(21.09, 25.17)	Z = −1.102	0.270
Family history of cancer	Yes No	71(47.02%) 80(52.98%)	31(41.33%) 44(58.67%)	χ^2^ = 0.654	0.419	37(47.44%) 41(52.57%)	22(44.90%) 27(55.10%)	χ^2^ = 0.078	0.780
Medication history	Yes No	23(15.23%) 128(84.77%)	13(17.33%) 62(82.67%)	χ^2^ = 0.165	0.684	17(21.79%) 61(78.21%)	−8(16.33%) 41(83.67%)	χ^2^ = 0.569	0.451
Number of pregnancies		2(1, 3)	2(2, 4)	Z = −1.575	0.115	2(2, 4)	2.5(2, 4)	Z = −0.791	0.429
Duration of lactation		16(6, 24)	18.83 ± 13.37	Z = −1.354	0.176	20(8, 24)	18.03 ± 14.31	Z = −0.811	0.417
Menopausal status	Yes No	14(9.27%) 137(90.73%)	14(18.67%) 61(81.33%)	χ^2^ = 4.075	0.044*	11(14.10%) 67(85.90%)	8(16.33%) 41(83.67%)	χ^2^ = 0.117	0.732
Menopausal age (postmenopausal)		48.50 ± 3.67	47.71 ± 2.53	T = −0.659	0.515	47.00 ± 2.73	49.45 ± 2.70	T = −1.950	0.068
Menarche age (premenopausal)		13(12, 14)	14(13, 15)	Z = −2.516	0.012*	14(13, 15)	14(13, 14)	Z = −0.045	0.964
Endocrine therapies	SERMs AIs OFS sequential therapy	61 11 60 24	27 11 26 6	χ^2^ = 5.440	0.142	30 11 24 13	19 5 16 9	χ^2^ = 0.448	0.930
Chemotherapy	Yes No	125(82.78%) 26(17.22%)	63(84%) 12(16%)	χ^2^ = 0.053	0.818	63(80.77%) 15(19.23%)	41(83.67%) 8(16.33%)	χ^2^ = 0.171	0.679
Number of radiotherapy		25(25, 30)	25(25, 26)	Z = −1.620	0.105	25(20, 30)	25(25, 27.75)	Z = −0.495	0.621
Targeted Therapy	Yes No	20(13.25%) 131(86.75%)	14(18.67%) 61(81.33%)	χ^2^ = 1.152	0.283	11(14.10%) 67(85.90%)	7(14.29%) 42(85.71%)	χ^2^ = 0.001	0.977
SAS		43.75(37.50, 48.75)	42.97 ± 9.62	Z = −0.891	0.373	42.5(37.5, 47.5)	42.5(38.13, 46.88)	Z = −0.446	0.655
SDS		44.59 ± 9.83	42.50(36.25, 51.25)	Z = −0.663	0.507	44.18 ± 11.1	42.5(37.5, 50.63)	Z = −0.295	0.768
FACT-B		107(94, 123)	108.60 ± 20.97	Z = −0.758	0.448	105.74 ± 19.91	107.82 ± 17.77	Z = −0.528	0.598

*Stands for statistical difference.

## References

[1] Sung H Ferlay J Siegel R Laversanne M Soerjomataram I Jemal A Global cancer statistics 2020: GLOBOCAN estimates of incidence and mortality worldwide for 36 cancers in 185 countries. *CA Cancer J Clin.* (2021) 71:209–49. 10.3322/caac.21660 33538338

[2] Allemani C Matsuda T Di Carlo V Harewood R Matz M Nikšić M Global surveillance of trends in cancer survival 2000-14 (CONCORD-3): analysis of individual records for 37 513 025 patients diagnosed with one of 18 cancers from 322 population-based registries in 71 countries. *Lancet.* (2018) 391:1023–75. 10.1016/S0140-6736(17)33326-3 29395269 PMC5879496

[3] Howlader N Altekruse S Li C Chen V Clarke C Ries L US incidence of breast cancer subtypes defined by joint hormone receptor and HER2 status. *J Natl Cancer Inst.* (2014) 106:dju055. 10.1093/jnci/dju055 24777111 PMC4580552

[4] Whisenant M Williams L Mendoza T Cleeland C Chen T Fisch M Identification of breast cancer survivors with high symptom burden. *Cancer Nurs.* (2022) 45:253–61. 10.1097/NCC.0000000000001019 34608052 PMC8964827

[5] Duarte A Hrynchak M Gonçalves I Quintela T Santos C. Sex hormone decline and amyloid β synthesis, transport and clearance in the brain. *J Neuroendocrinol.* (2016) 28:2587. 10.1111/jne.12432 27632792

[6] Biro E Kahan Z Kalman J Rusz O Pakaski M Irinyi T Cognitive functioning and psychological well-being in breast cancer patients on endocrine therapy. *In Vivo.* (2019) 33:1381–92. 10.21873/invivo.11615 31280234 PMC6689374

[7] Guidelines Working Committee of Chinese Society of Clinical Oncology. *Guidelines for diagnosis and treatment of breast cancer: 2020 edition [M].* Beijing: People’s Medical Publishing House (2020).

[8] Li J Gao W Sun L Bu Y Cao F The reliability and validity of Chinese version of cancer treatment function evaluation-cognitive function scale applied to breast cancer patients. *China J Pract Nurs.* (2015) 31:2554–6.

[9] Huo J Smith B Giordano S Reece G Shih Y. Comparative study of Cochran-Armitage trend test and linear regression in trend analysis of epidemiological rate. *Chin J Epidemiol.* (2017) 38:684–7. 10.1007/s10549-016-3832-x 27178333

[10] Rosenbaum P Rubin D. The central role of the propensity score in observational studies for causal effects. *Biometrika.* (1983) 70:41–55.

[11] Elze M Gregson J Baber U Williamson E Sartori S Mehran R Comparison of propensity score methods and covariate adjustment: evaluation in 4 cardiovascular studies. *J Am Coll Cardiol.* (2017) 69:345–57. 28104076 10.1016/j.jacc.2016.10.060

[12] Badhiwala J Karmur B Wilson J. Propensity score matching: a powerful tool for analyzing observational nonrandomized data. *Clin Spine Surg.* (2021) 34:22–4. 10.1097/BSD.0000000000001055 32804684

[13] Valera M Albert C Marcos J Larreategui Z Bori L Meseguer M. A propensity score-based, comparative study assessing humid and dry time-lapse incubation, with single-step medium, on embryo development and clinical outcomes. *Hum Reprod.* (2022) 37:1980–93. 10.1093/humrep/deac165 35904473

[14] Huang Y Wang D Lv W. Effects of treatment of traditional Chinese medicine on postoperative rehabilitation of tibial plateau fracture patients based on propensity score matching. *CJTCMP.* (2021) 36:4408–11.

[15] Wang J Lin J Chen L Chen Q Lin L Bao X Scandium and oral cancer: a case-control study based on propensity score matching. *J Environ Occup Med.* (2020) 37:421–6.

[16] Sun R. *To investigate the prognosis of postmenopausal breast cancer patients with neoadjuvant endocrine therapy based on propensity score matching method[D].* Changchun: Jilin University (2021).

[17] Pudkasam S Polman R Pitcher M Fisher M Chinlumprasert N Stojanovska L Physical activity and breast cancer survivors: importance of adherence, motivational interviewing and psychological health. *Maturitas.* (2018) 116:66–72. 30244781 10.1016/j.maturitas.2018.07.010

[18] Tjan-Heijnen V van Hellemond I Peer P Swinkels A Smorenburg C van der Sangen M Extended adjuvant aromatase inhibition after sequential endocrine therapy (DATA): a randomised, phase 3 trial. *Lancet Oncol.* (2017) 18:1502–11. 10.1016/S1470-2045(17)30600-9 29031778

[19] Gnant M Fitzal F Rinnerthaler G Steger G Greil-Ressler S Balic M Duration of adjuvant aromatase-inhibitor therapy in postmenopausal breast cancer. *N Engl J Med.* (2021) 385:395–405. 10.1056/NEJMoa2104162 34320285

[20] Chen X Li J Zhang J He X Zhu C Zhang L Impairment of the executive attention network in premenopausal women with hormone receptor-positive breast cancer treated with tamoxifen. *Psychoneuroendocrinology.* (2017) 75:116–23. 10.1016/j.psyneuen.2016.10.020 27815995

[21] Liao K Lin C Lai S. Nationwide case-control study examining the association between tamoxifen use and Alzheimer’s disease in aged women with breast cancer in Taiwan. *Front Pharmacol.* (2017) 5:612. 10.3389/fphar.2017.00612 28928665 PMC5591818

